# Scarring Alopecia in Localized Dystrophic Epidermolysis Bullosa: A Case Report and a Scoping Review

**DOI:** 10.7759/cureus.98811

**Published:** 2025-12-09

**Authors:** Pratiksha Patra, Sairekha Ravichandran, Nishit Patel, Paul Rodriguez-Waitkus, Wei-Shen Chen

**Affiliations:** 1 Internal Medicine, University of South Florida, Tampa, USA; 2 Dermatology, University of South Florida, Tampa, USA; 3 Dermatopathology, University of South Florida, Tampa, USA

**Keywords:** alopecia, dystrophic epidermolysis bullosa, epidermolysis bullosa, histopathology, scarring alopecia

## Abstract

Alopecia is a recognized complication of the epidermolysis bullosa (EB) group of blistering diseases, with diffuse and scarring alopecia seen in severe generalized EB. However, there is a dearth of literature characterizing alopecia in localized EB, with few reports of scarring and non-scarring alopecia in dystrophic EB (DEB).

In this report, we present a case of a 58-year-old female with an eight-year history of pretibial DEB and a documented heterozygous COL7A1 mutation, who presented to the dermatology clinic with improving skin fragility after five months of topical beremagene geperpavec therapy, now noting gradual hair thinning on the vertex scalp over one year. Clinical examination revealed decreased hair density on the vertex and frontal scalp, with scattered sclerotic papules, without significant perifollicular scale or erythema. Two punch biopsies were obtained for histologic vertical and horizontal sections, revealing superficial and mid-dermal fibrosis encircling hair follicles at the infundibulum and isthmus, numerous fibrous stelae, a superficial perifollicular lymphocytic infiltrate with scattered follicular dyskeratotic keratinocytes, and preservation of sebaceous glands. Together, these findings were consistent with scarring alopecia with shared features of DEB and lichen planopilaris (LPP). We highlight the importance of recognizing scarring alopecia as a complication of DEB, with discernment for concomitant inflammatory etiologies that may co-occur, especially in a patient with a COL7A1 mutation.

## Introduction

Epidermolysis bullosa (EB) is a group of inherited blistering disorders with varying defects of the basement membrane zone (BMZ) components, which can be broadly classified based on the level of skin cleavage: EB simplex (EBS, intraepidermal), junctional EB (JEB, epidermal-dermal interface or the BMZ), and dystrophic EB (DEB, below the lamina densa in the superficial dermis) [1]. DEB is caused by mutations in COL7A1, the gene encoding type VII collagen [2-4], a key component of anchoring fibrils that secure the epidermis to the dermis. In dominant DEB (DDEB), missense mutations lead to partially functional collagen VII and a milder phenotype, while in recessive DEB (RDEB), nonsense or frameshift mutations result in absent or nonfunctional collagen VII and a more severe clinical presentation [5-7].

In EB patients, alopecia is a known complication because the expression of BMZ components of the anagen hair follicle closely mimics that of the general interfollicular epidermis [8]. Chronic skin fragility, recurrent blistering, and prolonged wound healing in EB may contribute to hair loss through mechanisms that remain poorly understood, although existing literature has reported alopecia as nonspecific or absent in certain subtypes [6,9]. While diffuse, scarring alopecia is recognized in generalized and RDEB, there remains a gap in the literature characterizing alopecia in localized EB and DDEB, with even fewer reports of the histopathologic features in those instances.

In general, in the sparse literature, lichen planopilaris (LPP) has been anecdotally reported in patients with DDEB. LPP is a primary lymphocytic cicatricial alopecia characterized by an inflammatory infiltrate targeting the upper portion of the hair follicle, leading to follicular dropout and irreversible scarring. In this report, we present the case of LPP-like alopecia in a patient with DDEB, along with a scoping review of the current knowledge on EB-associated alopecia. Given the overlap in scarring fibrosis present in both primary inflammatory alopecia and EB-associated alopecia, close histopathologic discernment is needed to distinguish between the two diagnoses.

This case was previously presented as a meeting abstract at the 28th Joint Meeting of the International Society of Dermatopathology on March 6, 2025.

## Case presentation

**Figure 1:**
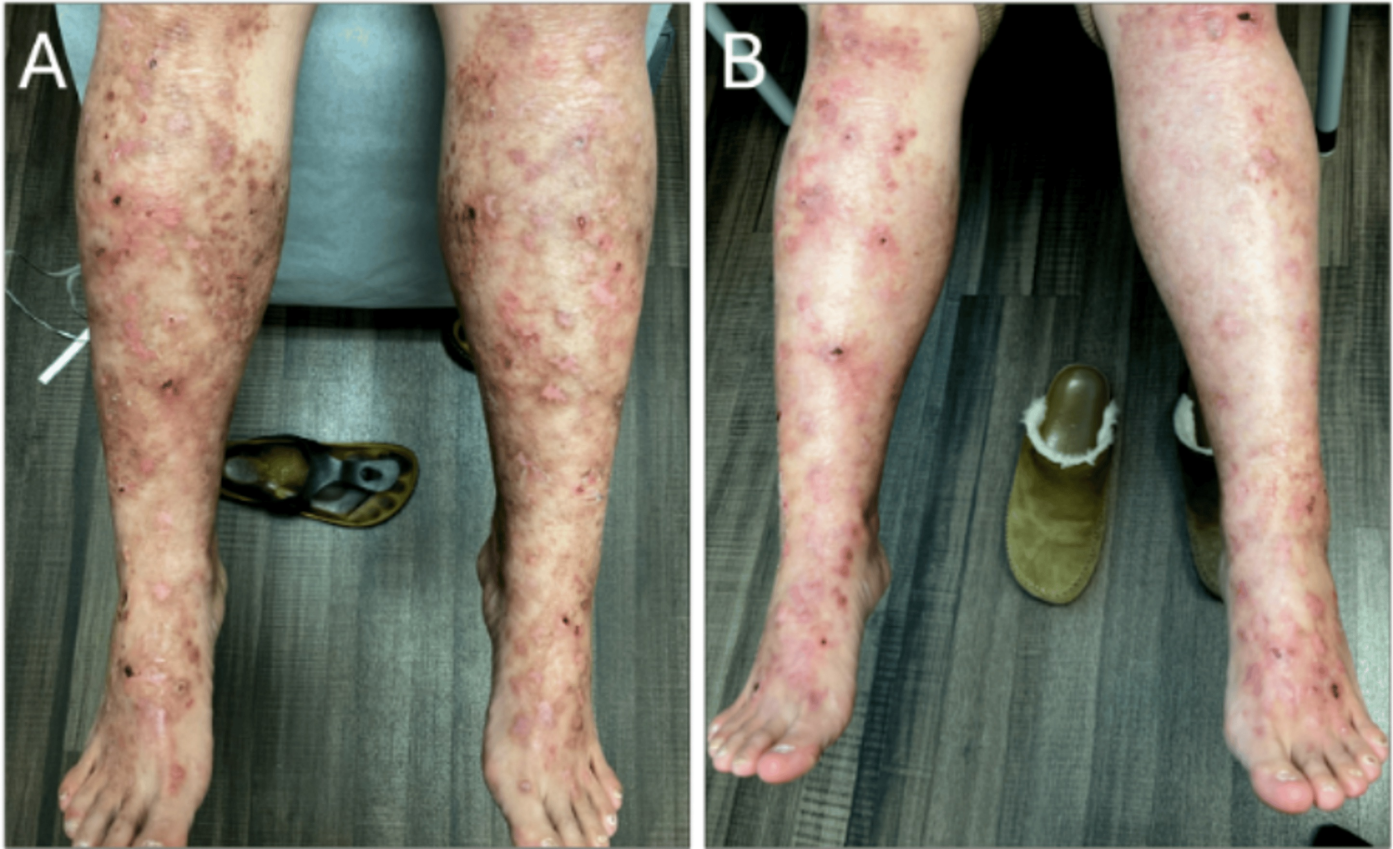
Improving pretibial dystrophic epidermolysis bullosa A) Initial presentation of the patient’s lower leg displaying vesicobullae, erosions, and pigmented scars from healed eruptions pre-treatment. B) Healing DEB lesions with fewer new blisters or erosions after six months of topical beremagene geperpavec therapy. DEB, dystrophic epidermolysis bullosa

**Figure 2:**
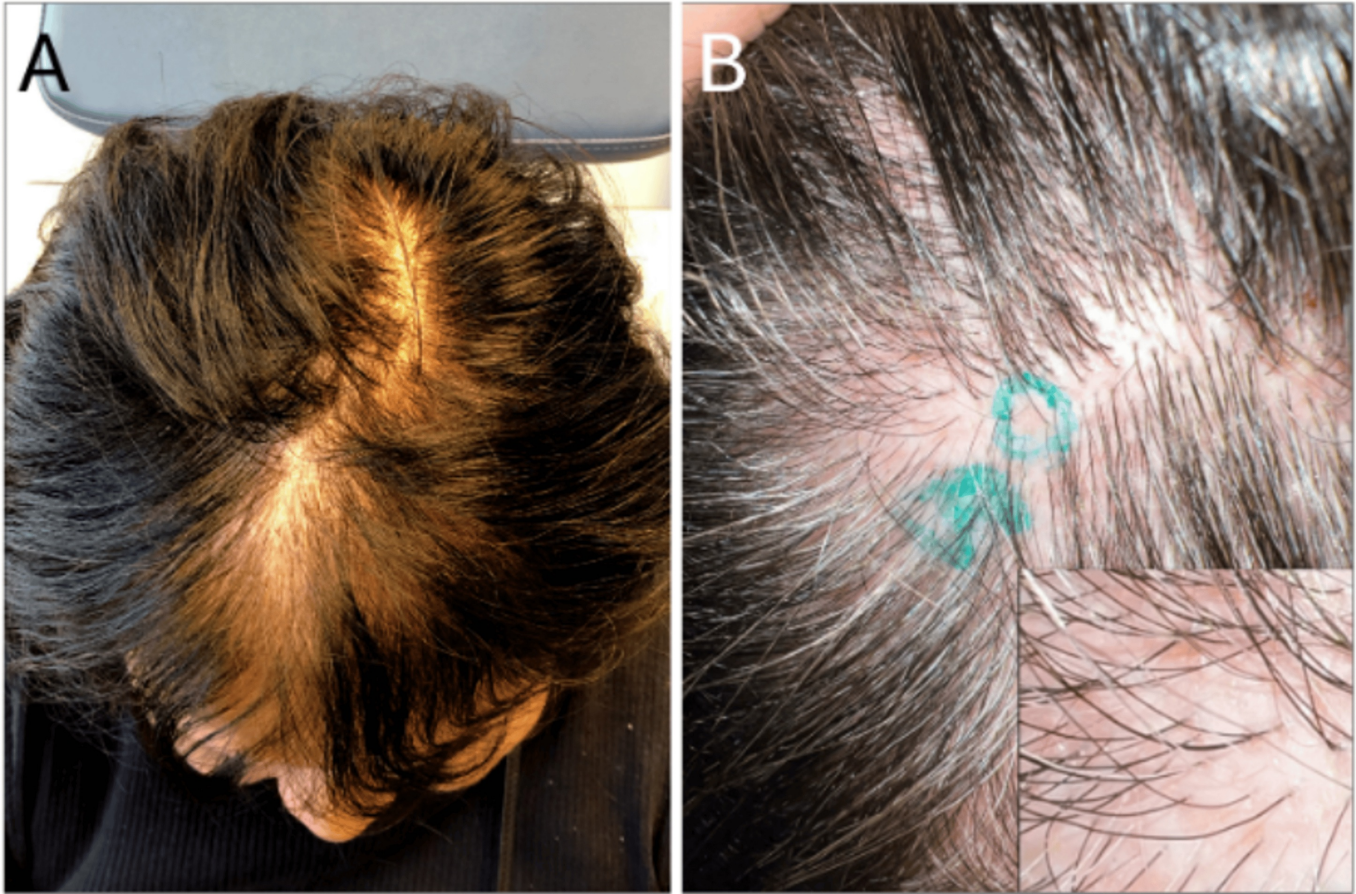
Diffuse hair loss, most notable on the vertex A) Hair density examination reveals visible frontal and vertex scalp thinning. B) Scalp examination reveals diffuse, mild scalp erythema and white sclerotic papules, without perifollicular scale or pigmentation on trichoscopy (not shown). Higher magnification is shown in the inset.

A 58-year-old female with an eight-year history of pretibial DEB, diagnosed by genetic testing revealing a heterozygous COL7A1 mutation, presented to our dermatology clinic with improving skin fragility after five months of topical beremagene geperpavec therapy (Figure 1), who now noted gradual hair thinning on the vertex scalp for one year. For context, pretibial DEB refers to a localized form of DEB affecting the shins, caused by mutations in the COL7A1 gene encoding type VII collagen, and beremagene geperpavec is a topical gene therapy designed to deliver functional COL7A1 to affected skin. The patient's DDEB diagnosis was initially achieved via skin punch biopsies from the lower leg, subject to manufacturer requirements for initiation of topical beremagene geperpavec therapy. She had immunomapping done when she was first diagnosed, and direct immunofluorescence ruled out pemphigus and pemphigoid. Regarding her scalp presentation, she denied associated pruritus, pain, or tenderness, and there was no history of oral or nail involvement. Prior treatment with topical minoxidil had failed. Clinical examination noted decreased hair density on the vertex and frontal scalp, with scattered white perifollicular dot-like areas but without total follicular dropout, erythema, or perifollicular hyperkeratosis, which are often seen in primary cicatricial alopecias (Figure 2). A clinical concern for a concurrent primary inflammatory alopecia, chiefly LPP, was noted, given the frontal distribution of the hair loss. Given her history of DDEB, secondary scarring alopecia related to her DDEB was also considered. 

**Figure 3:**
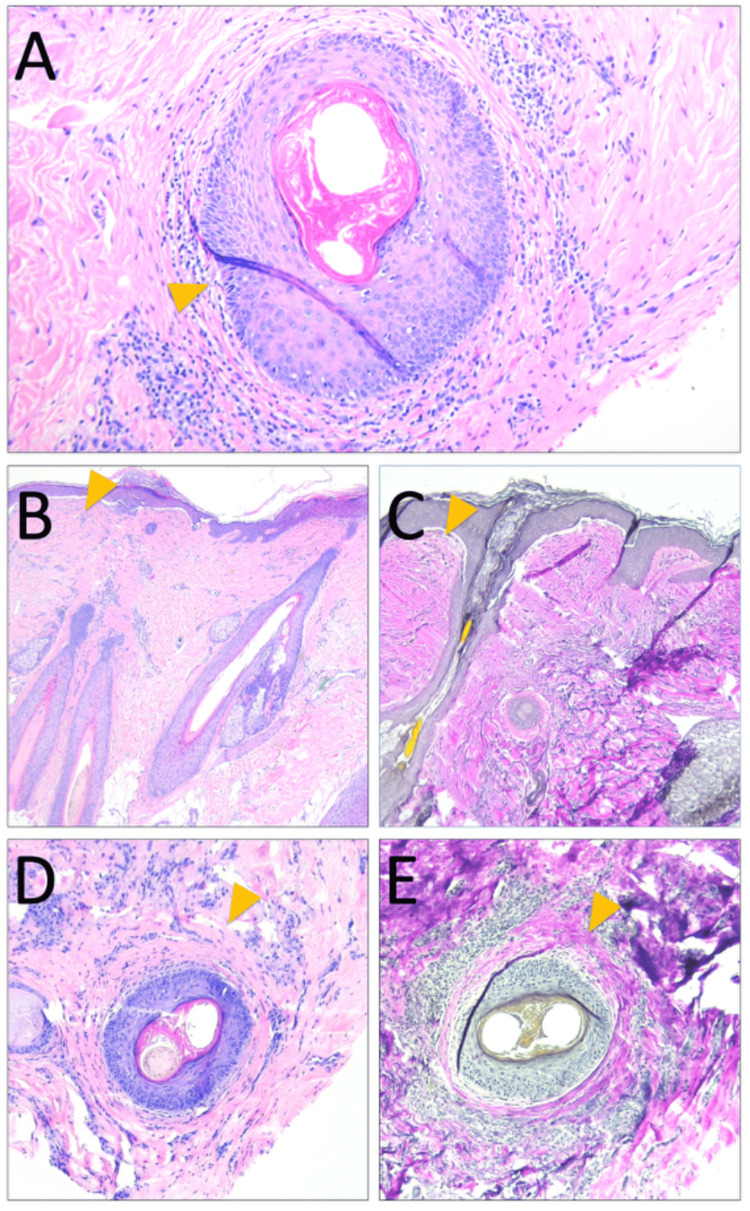
Histopathology of scarring alopecia in this DDEB patient A) Punch biopsy horizontal sections reveal perifollicular lymphocytic inflammation in the upper segment of the hair follicle. Rare dyskeratotic cells (yellow arrow) are seen. B) and D) Hematoxylin & eosin staining of vertical and horizontal sections shows mild perifollicular fibrosis. C) and E) Verhoeff-Van Gieson stain demonstrates diminished elastic fiber staining surrounding the upper-segment hair follicles on vertical and horizontal sections. DDEB, dominant dystrophic epidermolysis bullosa

Two 4-mm punch biopsies of the scalp were obtained for histologic vertical and horizontal sections. Histopathologic analysis revealed focal perifollicular lymphocytic infiltrate and concentric superficial and mid-dermal fibrosis encircling several hair follicles at the infundibulum and isthmus, consistent with scarring alopecia (Figure 3). In addition, there were numerous fibrous stelae and sebaceous glands diminished in size and number. No clefting or lichenoid inflammation at the dermal-epidermal interface was seen; however, rare dyskeratotic cells were present within the follicular epithelium (Figure 3A). Overall, the presence of perifollicular fibrosis and superficial lymphocytic infiltrate with focal follicular dyskeratosis suggested an inflammatory component. While the perifollicular inflammation was reminiscent of LPP, no significant interface changes or classic band-like junctional inflammation were identified. Moreover, the superficial dermis subjacent to the interfollicular epidermis appeared much more fibrosed than usual (Figures 3B-3C, arrowhead), lacking a well-defined wedge-shaped fibrosis surrounding the hair follicles, raising the possibility of scarring fibrosis secondary to this patient’s known DDEB. This is further evidenced by the patient's failed treatments with compounded tacrolimus and clobetasol after biopsy.

## Discussion

Methods of scoping review

A targeted scoping literature review, guided by PRISMA-ScR reporting elements, was performed to identify published reports of alopecia in EB. Searches were conducted in PubMed through August 2025, using combinations of EB subtypes (“junctional epidermolysis bullosa,” “dystrophic epidermolysis bullosa,” “recessive dystrophic epidermolysis bullosa,” “dominant dystrophic epidermolysis bullosa,” “JEB,” “RDEB,” “DDEB”) with alopecia-related terms (“alopecia,” “hair loss,” “cicatricial alopecia,” “scarring alopecia,” “inflammatory alopecia,” “non-scarring alopecia”). Reference lists of included articles were also screened to capture additional reports. The search yielded 30 articles in total, of which 13 met the inclusion criteria.

Eligible studies included case reports, case series, cohort studies, and registry analyses that described alopecia in genetically or clinically confirmed EB patients. Review articles were retained only when they provided patient-level information not otherwise captured in primary reports. Non-English publications, animal studies, and reports without an alopecia description were excluded. Each article was reviewed for EB subtype, number of patients, alopecia type (scarring vs. non-scarring), clinical and histopathologic features, and relevant notes.

Results

The findings were summarized and organized by EB subtype (JEB, RDEB, DDEB, DEB-unspecified, and EBS, for completeness) in Table 1. Among these published reports, several require clarification. Harris et al. [10] described the use of pigmented hair-thickening fibers in EB patients with alopecia; however, the reported diffuse hair loss was attributed to androgenetic alopecia and traction-related causes, rather than EB itself, and thus does not provide strong evidence of EB-associated alopecia. Similarly, Dillaha [11] reported a female infant initially thought to have DEB and nonspecific, non-scarring alopecia, but the case was subsequently reclassified as acrodermatitis enteropathica [12], with alopecia resolving after zinc supplementation, indicating that the hair loss was unrelated to EB. In contrast, Almaani et al. [13] presented a case of DDEB-pruriginosa complicated by LPP-like scarring alopecia, confirmed with biopsy findings of supra-isthmic lymphocytic inflammation and vague perifollicular fibrosis; this represents the only reported case of EB associated with a secondary inflammatory alopecia and is very similar to the histologic findings in our patient with DEB.

**Table 1:** Published cases describing alopecia in epidermolysis bullosa EB, epidermolysis bullosa; DEB, dystrophic epidermolysis bullosa; JEB, junctional epidermolysis bullosa; GABEB, generalized atrophic benign epidermolysis bullosa; RDEB, recessive dystrophic epidermolysis bullosa; DDEB, dominant dystrophic epidermolysis bullosa; EBS, epidermolysis bullosa simplex; LPP, lichen planopilaris

EB Subtype	Scarring/Inflammatory Alopecia	Non-scarring/Non-inflammatory Alopecia
JEB (GABEB, generalized intermediate, localisata, nH-JEB/COL17A1)	Harris et al. (2016) [10]: 2 generalized intermediate JEB patients had diffuse scarring alopecia (concomitant processes: traction in 1, androgenic in 1)	Floeth et al. (1998) [14]: 3 GABEB patients with alopecia of varying severity; 1 JEB-localisata patient with normal hair; Mazzanti et al. (1998) [15]: 2 of 4 GABEB patients had atrophic alopecia; Hintner and Wolff 1982 [16]: 8 GABEB patients consistently described with atrophic alopecia [16]; Swensson and Christophers (1998) [17]: 2 GABEB siblings with atrophic alopecia; Bauer and Lanschuetzer (2003) [18]: Patients with non-Herlitz JEB due to COL17A1 mutations consistently had atrophic alopecia as a distinct feature
RDEB	Matard et al. (2022) [19]: 30 of 125 patients (24%) had folliculitis decalvans, a neutrophilic scarring alopecia [19]; Harris et al. (2016) [10]: 1 RDEB-inversa patient was documented with alopecia, shown photographically	-
DDEB, pruriginosa variant	Rivitti-Machado et al. (2018) [20]: 1 EB-pruriginosa patient with folliculitis decalvans (neutrophilic scarring alopecia); Almaani​​​​​​​ et al. (2009) [13]: 1 EB-pruriginosa patient with LPP-like inflammatory scarring alopecia, confirmed histologically	-
DEB (unspecified)	Xie et al. (2019) [6]: Across 29 patients (from 15 reports), scarring alopecia was described in all DEB cases; Tosti et al. (2010) [8]: Cicatricial alopecia described in EB patients, without subtype breakdown	Dillaha (1952) [11] and Dillaha et al. (1953) [12]: 1 female infant reported with DEB and alopecia, though later questioned to instead be acrodermatitis enteropathica)
Unspecified or Other EB (EBS)	-	Xie et al. (2019) [6]​​​​​​​: Nonscarring alopecia was reported in most EBS patients [6]; Tosti et al. (2010) [8]: Alopecia universalis was reported in EB, without subtype specified [8]

Discussion

Alopecia is a known manifestation of several inherited forms of EB, including JEB and DEB. Both conditions can impair the function of follicular stem cell niches, contributing to the disruption of hair follicle regeneration [5]. In DEB, mutations in the COL7A1 gene disrupt type VII collagen, which leads to skin fragility in the sublamina densa. It has also been shown to disrupt follicular stem cell niches and impair follicular regeneration, which could contribute to the development of scarring alopecia in DEB [7]. In JEB, autosomal recessive mutations in genes encoding components of laminin-332 (LAMA3, LAMB3, LAMC2) or COL17A1 (which encodes type XVII collagen) also disrupt dermo-epidermal adhesions, but at the lamina lucida [21,22]. Persistent erosions in generalized severe (Herlitz) and intermediate JEB lead to scarring alopecia because, like in DEB, follicular regeneration is impaired [21]. Thus, while genetic mutations differ between JEB and DEB - affecting different collagen proteins and areas of the epidermal-dermal junction - both disorders may present with similar clinical features of hair loss. Furthermore, chronic microtrauma from epidermal fragility and blistering may trigger a secondary inflammatory response, leading to the perifollicular fibrosis observed in our patient [6].

The resemblance of our patient’s histopathologic findings to LPP raises important considerations regarding the pathogenesis of alopecia in localized DEB. One possibility is that localized DEB predisposes to secondary scarring alopecia through chronic inflammation, repeated trauma, and abnormal wound healing. Alternatively, the upper follicular inflammation and fibrosis observed may be intrinsically linked to the COL7A1 mutation and basement membrane dysfunction in DEB, rather than representing a bona fide inflammatory alopecia, such as LPP. Further distinguishing LPP from DEB-associated alopecia requires assessing patterns of fibrosis and inflammation. LPP typically presents with concentric, V-shaped perifollicular fibrosis, whereas our patient displayed both concentric and horizontal fibrosis, a pattern that may be influenced by the underlying structural abnormalities of DEB. Additionally, while LPP is associated with dense lymphocytic inflammation surrounding the epidermal and follicular epithelial interfaces and ultimately obliterating the infundibular and isthmus levels of the follicular epithelium, our patient’s inflammation was more along the superficial infundibular follicle and lacked the dense, band-like inflammation or epidermal interface changes characteristic of classic LPP. Notably, the distribution of type VII collagen corresponds to the infundibulum, the same site primarily affected by the lymphocytic follicular inflammation in LPP, raising the possibility that LPP-like changes in DEB-related alopecia could be associated with underlying BMZ defects [23]. The presence of dyskeratosis in our patient’s biopsy further complicates the distinction between these entities, as dyskeratotic keratinocytes can be seen in both LPP and EB-related inflammatory responses [5,13,23]. Given this overlap, our case provides an opportunity to explore whether inflammatory alopecias, like LPP, share mechanistic features with DEB, particularly in the setting of BMZ dysfunction.

Notably, Almaani et al. [13] reported a similar case of DDEB-pruriginosa complicated by LPP-like scarring alopecia, confirmed with biopsy findings of supra-isthmic lymphocytic inflammation and perifollicular fibrosis. This case was interpreted as a separately acquired LPP. Given the similarities between that case and our patient, this case may represent another example within the spectrum of DDEB-associated scarring alopecia and also sways against the argument that our patient had a treatment-related inflammatory alopecia. Future considerations may include exploring whether topical therapies used on DEB-affected skin could offer any benefit to the scalp, as well as evaluating antifibrotic agents to support hair growth.

## Conclusions

In summary, we highlight the importance of recognizing alopecia as a complication of localized DEB and advocate for careful histologic assessment to distinguish it from primary inflammatory alopecia. Although causality cannot be established, the temporal onset of alopecia following initiation of topical beremagene geperpavec raises the possibility that treatment-related inflammation may have contributed to the clinical presentation. We recommend scalp surveillance and early biopsy in DEB patients reporting hair thinning. We further posit that inflammation of the upper segment of the hair follicle, with surrounding follicular fibrosis - changes resembling LPP - may be intimately associated with the underlying COL7A1 defect of DEB, rather than being diagnostic features of a distinct, concomitant inflammatory primary alopecia. This is potential evidence of a novel variant associated with DEB. Diagnostic uncertainty in such cases highlights the need for additional investigative strategies, including longitudinal clinical follow-up, correlation with treatment response, and ancillary studies, such as immunohistochemistry or molecular analyses, to better delineate DEB-associated changes from true inflammatory alopecias. Future studies are needed to further investigate the mechanisms of alopecia in DEB and determine whether targeted therapies, such as anti-inflammatory or anti-fibrotic treatments, may be beneficial in affected patients.
